# The effect of a single-session heart rate variability biofeedback on attentional control: does stress matter?

**DOI:** 10.3389/fpsyg.2023.1292983

**Published:** 2023-11-16

**Authors:** Berenike L. Blaser, Mathias Weymar, Julia Wendt

**Affiliations:** ^1^Department of Biological Psychology and Affective Science, Faculty of Human Sciences, University of Potsdam, Potsdam, Germany; ^2^Faculty of Health Sciences Brandenburg, University of Potsdam, Potsdam, Germany

**Keywords:** attention, self-regulation, heart rate variability, biofeedback, cognitive control, stress, vagal tone, slow-paced breathing

## Abstract

**Introduction:**

Vagally mediated heart rate variability is an index of autonomic nervous system activity that is associated with a large variety of outcome variables including psychopathology and self-regulation. While practicing heart rate variability biofeedback over several weeks has been reliably associated with a number of positive outcomes, its acute effects are not well known. As the strongest association with vagally mediated heart rate variability has been found particularly within the attention-related subdomain of self-regulation, we investigated the acute effect of heart rate variability biofeedback on attentional control using the revised Attention Network Test.

**Methods:**

Fifty-six participants were tested in two sessions. In one session each participant received a heart rate variability biofeedback intervention, and in the other session a control intervention of paced breathing at a normal ventilation rate. After the biofeedback or control intervention, participants completed the Attention Network Test using the Orienting Score as a measure of attentional control.

**Results:**

Mixed models revealed that higher resting baseline vagally mediated heart rate variability was associated with better performance in attentional control, which suggests more efficient direction of attention to target stimuli. There was no significant main effect of the intervention on attentional control. However, an interaction effect indicated better performance in attentional control after biofeedback in individuals who reported higher current stress levels.

**Discussion:**

The results point to acute beneficial effects of heart rate variability biofeedback on cognitive performance in highly stressed individuals. Although promising, the results need to be replicated in larger or more targeted samples in order to reach stronger conclusions about the effects.

## 1. Introduction

“Take a deep breath” is an idiom commonly used in everyday life. Most people seem to understand that breath control has some kind of connection to one’s mental state. Indeed, the goal of these breathing exercises is usually to refocus attention away from salient stimuli toward more relevant targets, such as interrupting anxious thought loops before an exam or bringing the attention back from unfocused distraction. Research shows consistent associations between this skill of top-down regulation and the physiological correlate of vagally mediated heart rate variability (vmHRV), an index of parasympathetic activity (Thayer and Lane, 2000; Laborde et al., 2023). Critically, vmHRV parameters are strongly influenced by breath (Vaschillo et al., 2006). This influence is driven by respiratory sinus arrhythmia (RSA), the phenomenon where heart rate increases with inhalation and decreases with exhalation (Eckberg, 1983; Berntson et al., 1993). These sinus wave-like fluctuations of heart rate are produced by phasic vagal input to the sinoatrial node during exhalation (Vaschillo et al., 2006). RSA is largely vagally mediated (Karemaker, 2022), and the quantifications of the contribution of RSA to heart rate [although not the magnitude of the RSA itself, see Grossman and Taylor (2007), Kollai and Mizsei (1990)] are interpreted as the extent of cardiac parasympathetic activation. In heart rate variability biofeedback (HRV BFB) interventions, RSA can be maximized through slow-paced breathing, which increases HRV both during and after the intervention (Laborde et al., 2022a). Practicing this slow-paced breathing over a longer period of time (e.g., 4 weeks) has been shown to have a wide range of positive emotional and cognitive effects (Goessl et al., 2017; Lehrer et al., 2020; Pizzoli et al., 2021).

Two recent meta-analyzes by Holzman and Bridgett (2017) and Zahn et al. (2016) found consistent, albeit small associations between vmHRV measures at rest and performance on different laboratory self-regulation tasks. A theoretical framework for this association has been proposed by Thayer and Lane (2000) in their model of neurovisceral integration. The authors suggest vmHRV as a peripheral marker for the capacity as well as a reciprocal functional part of the central autonomic network (CAN). Thayer and Lane (2009) suggest that the reciprocal inhibitory connectivity of the ventromedial prefrontal cortex (vmPFC) and the amygdala is the core mechanism of this complex system, which coordinates behavioral, cognitive and physiological self-regulation. Thayer and Lane (2000) argue that vmHRV can be viewed as an indicator of the extent of the influence the higher processing structures such as the vmPFC have on brainstem and autonomic activity. This reflects the organism’s capacity to inhibit automatic responses and instead react flexibly to environmental demands. In their vagal tank theory, Laborde et al. (2018) expand on the neurovisceral integration framework. The authors propose that cardiac vagal control (vmHRV) reflects self-regulatory resources, which can be depleted or replenished like a tank. While resting vmHRV is positively associated with self-regulation, vmHRV reactivity’s association with self-regulation depends on the level of activity and stress. In high activity/stress situations, a larger reduction in vmHRV is seen as adaptive, while in low activity/stress situations, a lower drop is better for self-regulatory performance. Self-regulation manifests itself in multiple domains. Although not statistically significant, both meta-analyzes on resting vmHRV and self-regulation (Zahn et al., 2016; Holzman and Bridgett, 2017) have observed that these associations are larger in the attentional control domain. One way of assessing attentional control is via the revised attention network test (ANT-R, Fan et al., 2009). The ANT-R differentiates between the three networks of attentional processes proposed in the attentional network theory by Posner and Petersen (1990). The Alerting Network is the network that sets the system into a general, vigilant arousal state enabling faster reaction times once action is needed. The Orienting Network is a system of structures that enables the efficient and rapid selection of the correct modality and location from which sensory input should be primarily processed. In doing so, salient information that is not relevant for goal attainment is suppressed. Petersen and Posner (2012) describe the Executive Network as a system to provide focal attention, the limited awareness of relevant information which inhibits the processing of other input and enables the complex neuro-structural activation system that comprises consciousness. In comparison to the Alerting and Executive Networks, the Orienting Network, which is assessed through a cueing paradigm and specifically requires active employment of goal-directed self-regulation, has been closely linked to vmHRV measures at rest [medium to large associations (Pearson’s R between −0.3 and −0.55); Quintana et al. (2017), Ramírez et al. (2015), Sørensen et al. (2019)]. Additionally, active engagement of the Orienting Network is associated with activation in the ventral PFC (Petersen and Posner, 2012), which plays a central role in the CAN proposed by Thayer and Lane (2000).

All parts within Thayer and Lane (2009) CAN are assumed to be bidirectional, which implies that the manipulation of vmHRV can have modulational effects on self-regulatory capacity. One way to improve vmHRV is through HRV BFB training, in which the individual receives visual feedback on momentary vmHRV and learns to influence it via breathing rhythm. A slow-paced breathing frequency is set at 0.1 Hz or at an individual resonance frequency between 0.07–0.12 Hz (Lehrer et al., 2013), which has been shown to maximize (individual resonance frequency) or at least strongly increase (0.1 Hz) RSA (Vaschillo et al., 2006). And indeed, research suggests that the effects of HRV BFB are almost exclusively driven by slow-paced breathing rather than the visual feedback of momentary HRV (Laborde et al., 2022c).

It has been suggested that, among other interventions, particularly abdominal slow-paced breathing at around 0.1 Hz increases vmHRV through the approach of resonance of the respiration-heart beat phasic relationship and the baroreflex (Lehrer and Gevirtz, 2014). A recent literature review elaborates on this by explaining how the temporal coherence between respiratory, blood pressure, and cardiac phases offers the ideal timing for a complete release of acetylcholine, which orchestrates the reactive drop in heart rate during exhalation, and its subsequent hydrolysis (Sevoz-Couche and Laborde, 2022). This, in turn, leads to an enhanced baroreflex. HRV BFB has been found not only to enhance baroreflex gain during training but also to produces long-term increases in baroreflex gain at rest (Lehrer et al., 2003). The altered autonomous activity can then affect higher cognitive processing levels through afferent pathways. Lehrer and Gevirtz (2014) describe one of the most important among these pathways to be the baroreflex afferent input during exhalation to the amygdala via the nucleus of the solitary tract, where sensory signals from the baroreceptors are integrated and processed and projections branch extensively into structures of the CAN (Henderson et al., 2004). More recent research has found additional effects of a complementary afferent input to the nucleus of the solitary tract via afferents from slowly adapting pulmonary stretch receptors, which increases sympathetic activity during prolonged inhalation while their activity is terminated during prolonged exhalation (Noble and Hochman, 2019).

In addition, HRV BFB may stimulate the afferent vagal pathway (Lehrer and Gevirtz, 2014), which also projects into all structures of the CAN and can therefore influence higher cognitive processing levels as proposed in the theory of neurovisceral integration (Thayer and Lane, 2009). Through these afferent pathways projecting into the CAN, the non-adaptive default threat response as proposed by Thayer and Lane (2000) can be altered to ensure more adaptive regulatory processes. Gerritsen and Band (2018) propose that this respiratory vagus nerve stimulation is the driving factor behind the many benefits of contemplative practices.

Furthermore, the interplay between the afferent input from the pulmonary stretch receptors and baroreceptors do not only impact CAN activity but also produces synchronized cortical rhythms in the same frequency as the slow-paced breathing (Noble and Hochman, 2019). These slow global potentials could potentially interact with other networks involved in stress and memory, including the default mode network. Mental effects of the concentrative practice in HRV BFB as well as direct cortical pathways via activation of the olfactory bulb might be additional elements (Lehrer and Gevirtz, 2014).

Based on the proposed mechanisms of HRV BFB (Lehrer and Gevirtz, 2014), it is not clear which of the effects of HRV BFB are immediate and which develop over the course of long-term training. Several meta-analyzes (Goessl et al., 2017; Lehrer et al., 2020; Pizzoli et al., 2021) demonstrate the beneficial long-term effects of HRV BFB on cognitive and emotional states, possibly by way of increased general activity as well as functional connectivity of and within the CAN (Schumann et al., 2021). Tinello et al. (2022) found mixed results in a systematic review of HRV BFB effects on executive functions with about half of the included studies reporting beneficial effects. The authors reported effects especially in the domain of attention and inhibition and in vulnerable populations such as samples experiencing high stress or clinical samples. However, it remains unclear whether the acute increase in CAN activity due to afferent vagal activation is sufficient to observably affect cognitive and emotional measures.

A small number of studies, however, already point to immediate beneficial effects in terms of stress relief. One study found acute anxiety-reducing and calming effects of HRV BFB in a sample of students with high perceived stress levels (Meier and Welch, 2016). Similarly, another study found that HRV BFB acutely reduces the excitability of motoneurons in the medulla (Pagaduan et al., 2021). Prinsloo et al. (2013a) studied a small group of men (*N* = 18) in managerial positions who rated high in perceived life stress as well as current work stress, and found acute increases in self-reported relaxation, energized positive feelings and mindfulness and decreases in anxiety (Prinsloo et al., 2013b). Additionally, they showed changes in electroencephalography (EEG) signals both during and after the intervention, which reflected increased relaxation and attention (Prinsloo et al., 2013c). Furthermore, lower levels of salivary alpha-amylase were found in participants who completed an HRV BFB session after a stress-inducing laboratory task compared to a control group, but no difference in cortisol levels or self-reported stress, indicating differential effects on different measures of stress (Hunter et al., 2019). A last study employing a one session slow-paced breathing intervention additionally showed changes in EEG frequency activity, i.e., increases in alpha-band activity and decreases in beta-band activity in areas critical to stress regulation as revealed by source localization (Sherlin et al., 2010).

Additionally, a handful of studies suggest an acute improvement effect of single-session HRV BFB on cognitive outcomes. In the study elaborated on in the previous paragraph, better performance on a modified Stroop task was also observed after the BFB intervention compared to a control condition (Prinsloo et al., 2011, 2013a). Similarly, better performance in Stroop tasks was found after a one-session slow-paced breathing intervention in a population of adults who reported high stress levels (Sherlin et al., 2010), and in a population of athletes who underwent a physical exhaustion protocol (Laborde et al., 2019). Another study also reported similar findings as well as improved performance in an operation span task after a slow-paced breathing intervention compared to a control condition (Laborde et al., 2022b). Hoffmann et al. (2019) investigated the effects of slow-paced breathing on the performance in a flanker task. The results showed no significant effect on task performance, but there was an increase in the amplitude of the error-related negativity component of the event-related potential, which has been interpreted as indicating increased attention to performance accuracy.

Taken together, these results point to acute effects of alleviating stress and improving cognitive outcomes. Most of the above-mentioned studies, however, focused on samples that rated high in baseline stress levels or underwent a stress-inducing protocol. While high self-reported stress might make individuals more susceptible to the BFB intervention, this question has not explicitly been examined yet.

To investigate the acute effects of HRV BFB on attentional control and whether or not stress is a moderating factor, we conducted a laboratory study that tests the effects of a single-session HRV BFB on the ANT-R. As a manipulation check in this study, we hypothesize that vmHRV indexed by the root mean square of successive differences (RMSSD) is higher during an HRV BFB intervention compared to paced normoventilation during a control condition (Hypothesis 1). Secondly, we aim to replicate the positive association between resting vmHRV and attentional control (Hypothesis 2). We furthermore hypothesize acute improvements in the Orienting Network score after an HRV BFB session compared to a control condition (Hypothesis 3). Lastly, we postulate that the effect of HRV BFB on the Orienting score of the ANT-R is moderated by the individual stress level, as indicated by a self-report questionnaire on current stress (Hypothesis 4).

## 2. Materials and methods

### 2.1. Participants

Sixty participants were recruited from the population of students at the University of Potsdam through flyers on campus as well as the online recruiting platform for study participants of the cognitive sciences (Sona Systems, https://www.sona-systems.com). One person dropped out after the first session and three had to be excluded due to incomplete data, leaving a sample size of 56 (age = 23.1 ± 3.4 years, 75% women, 23% men, 2% diverse), see Table 1. Informed consent was given by all participants after being given both verbal and written information about the study. The study protocol was approved by the ethics committee of the University of Potsdam (proposal No 15/2021).

**Table 1:** Sample characteristics (*N* = 56).

Variable	Mean	SD	Min	Max
Age in years	23.1	3.41	19	30
BMI	21.4	2.4	18.4	29
Gender	75% female; 23% male, 2% diverse			
RMSSD baseline in ms	44.4	31.5	12.2	207.3
RMSSD gain from BFB in ms	20.3	17.1	−42.3	56
ANT-R Alerting network score	37.5	32.3	−48.7	127.8
ANT-R Orienting network score	95	37.6	10.1	190.8
ANT-R Executive network score	122	32.1	49	221.4
ANT-R Orienting-Executive interaction score	18.8	37.7	−89.1	127
ANT-R Alerting-Executive interaction score	−1	52.2	−148.8	143.2
Perceived stress scale score	27.9	6.1	14	41

SD, standard deviation; Min, minimum; Max, maximum; BMI, body-mass-index; RMSSD, root mean square of successive differences, BFB, biofeedback; ANT-R, revised attention network test.

Following recommendations by Laborde et al. (2017), individuals were excluded who took medication that alters autonomic functioning, had a chronic or acute disease associated with altered autonomic functioning or were pregnant. Competitive athletes were also excluded, as athletes show systematically altered HRV (Da Silva et al., 2015). Since vmHRV varies across the lifespan (Voss et al., 2012), in order to achieve a homogenous sample participants younger than 18 years and older than 30 years were excluded.

The sample size was calculated based on the effect size of HRV BFB on the RMSSD, which is around *d* = 1.6 (Laborde et al., 2022c). The Orienting component of the ANT-R shows a correlation between 0.3 and 0.55 with vmHRV at rest (Ramírez et al., 2015; Quintana et al., 2017; Sørensen et al., 2019). Assuming conservatively that about half of the shared variance of the lowest value (covariance = r^2^ = .3^2^/2) might respond to the intervention leads to an estimated effect size of d = 1.6 * sqrt(.3^2^/2) = 0.34. To observe this main effect in a within-subject design, a G*Power analysis (Faul et al., 2007) revealed a necessary sample size of *N* = 55. To account for dropouts and data trimming, 60 participants were tested.

### 2.2. Testing procedure

Participants were tested in two different sessions exactly 1 week apart, at the same time of day between 9 am and 7 pm. Before signing up for the testing sessions, participants filled out an online questionnaire. The online questionnaire screened for exclusion criteria and assessed current perceived stress levels (German version of the Perceived Stress Scale – PSS; Klein et al., 2016) as well as information known to be associated with vmHRV, namely age, gender (Zhang, 2007), body-mass-index (BMI, Molfino et al., 2009), and habitual use of caffeine (Koenig et al., 2013), nicotine and alcohol (Koskinen et al., 1994).

Following the recommendations of Laborde et al. (2017), participants were asked not to drink alcohol or do intense physical training, and to follow their normal sleep routine and take note of the time they fell asleep and woke up 24 h before the sessions. Furthermore, they were instructed not to drink caffeinated drinks or have a meal 2 hours before each session.

At the beginning and end of each session, the RMSSD at rest was measured to obtain a baseline RMSSD. Participants had been sitting down for at least 15 min before the measurement. The measurement was taken in a sitting position. Participants were instructed to sit comfortably, place their feet side by side on the floor, close their eyes and were told that they do not have to pay attention to anything in particular. Following the recommendations by Laborde et al. (2017), the measurement had a duration of 5 min.

After the initial RMSSD assessment, a one-minute threshold assessment followed in each session. Secondly, participants completed a 5-min HRV biofeedback training in one session and a comparative control condition in which normoventilation (NV) was applied in the other session.

**Figure 1:**
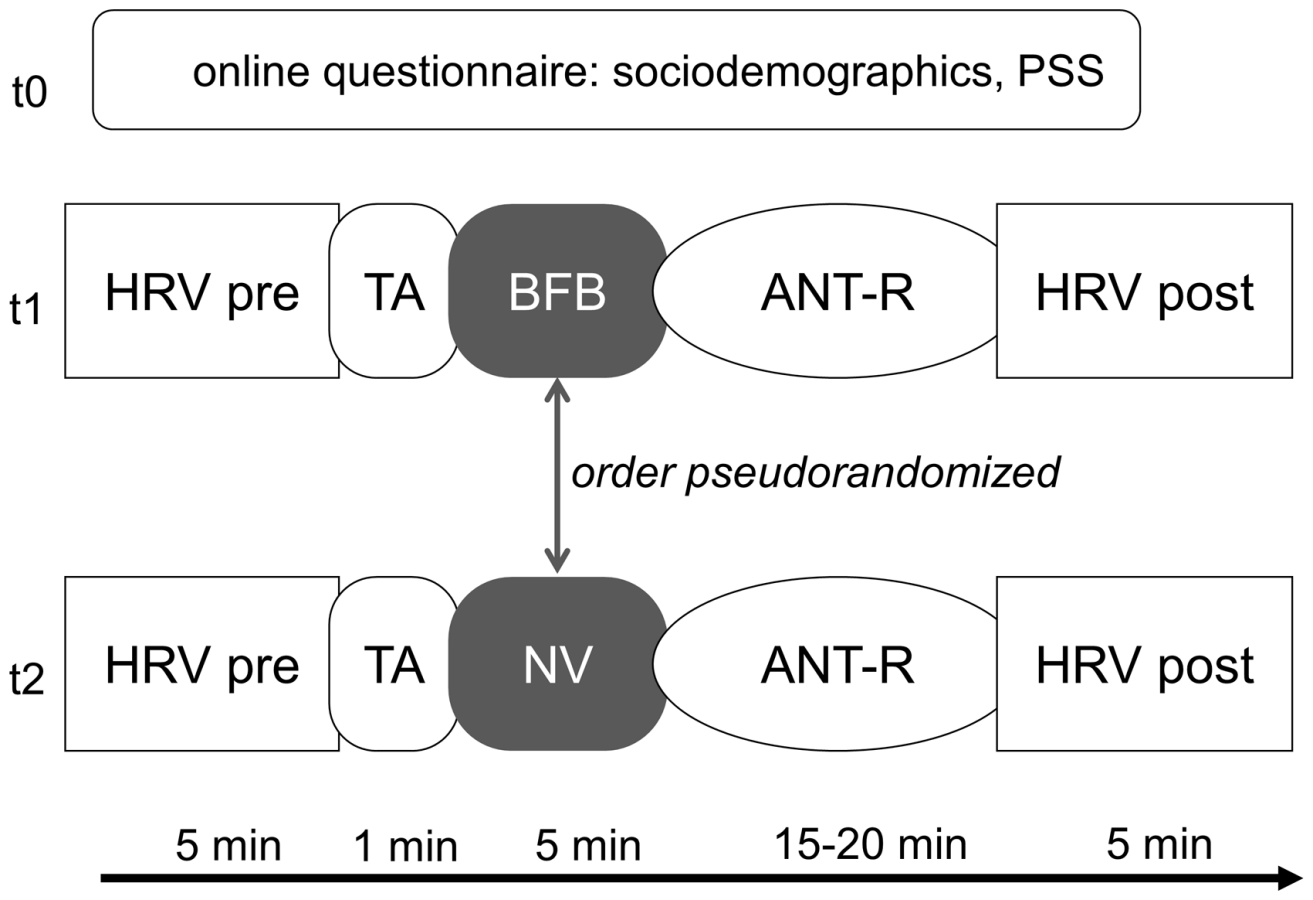
Testing procedure. The order of BFB and control condition is randomized within sociodemographic groups. PSS, perceived stress scale; HRV, heart rate variability; TA, threshold assessment; BFB, biofeedback; NV, normoventilation; ANT-R, attention network test-revised. Online questionnaire was filled out before sign up, t1 and t2 were exactly 1 week apart at the same time of day.

Each participant was tested in both NV and BFB conditions. The order in which the participants received BFB and NV was pseudo-randomized. Since gender effects may be expected due to prior research showing differential RMSSD between those groups (Sammito and Böckelmann, 2016), the order of BFB and NV conditions was balanced among biological men and women. Additionally, an approximation of an age median split was applied before the testing, based on prior samples in studies of our group (</≥ 23 years of age) (e.g., Wendt et al., 2019; Szeska et al., 2020). Within each of the four subgroups (women <23 y/o, women ≥23 y/o, men <23 y/o, men ≥23 y/o), condition order was balanced. After the breathing intervention, the ANT-R was completed followed by a second RMSSD assessment at rest (see Figure 1).

### 2.3. Heart rate variability assessment, biofeedback and normoventilation

Resting RMSSD and biofeedback threshold assessments as well as HRV biofeedback (and the control condition) were conducted using the BioSign soft- and hardware (“HRV-Scanner”; Biosign®, D-85570, Ottenhofen, Germany). Heart rate was measured by a one-lead electrocardiogram (ECG) through two surface sensors attached to the right and left wrists of the participant. The device works with a sampling rate of 500 Hz and a 16-bit resolution.

Artifacts and abnormal beats were filtered in a two-step process following the protocol outlined in the HRV-Scanner software documentation (BioSign GmbH, 2023). First, the HRV-Scanner software automatically marked areas of the heart rate curve that included implausible changes in heart rate through the division of the heart rate curve into small segments and a subsequent scan of each segment. This process is based on an algorithm patented by the BioSign^®^ company that identifies outliers in a Poincaré plot, in which each RR interval is plotted against the previous RR interval.

Working with these recognized areas of possible disturbances, in the second step the R-spike recognition was manually assessed and corrected, and artifacts, alterations of the electric signal not related to the electrical activity of the heart due to movement or the like, were removed. After the two-step process the data quality was excellent, with less than 0.1% artifacts per measurement on average. Only 7% of the heart rate assessments had a measurement quality of less than 100%, of which the lowest was 96.6%.

#### 2.3.1. HRV baseline assessment

We focused on the RMSSD as a measure of vmHRV (Penttilä et al., 2001). The high frequency (HF) component of power spectral analyzes, which has also been shown to assess cardiac parasympathetic activity (Penttilä et al., 2001), is not used in this study for the following reasons. The HF is defined as the power of the frequency band between 0.15 and 0.40 Hz. This frequency band is based on normoventilation at a rate of 9–24 breathing cycles per minute. Heart rate oscillations in this frequency reflect RSA, which is largely driven by vagal activity. However, during the BFB intervention applied in this study, the paced breathing frequency was set at 0.1 Hz. A maximal peak at this frequency in power spectral analysis indicates maximal RSA. The RSA peak is therefore not inside the HF band and can consequently not be quantified by this parameter. In fact, during slow-paced breathing, the vagal contribution to heart rate oscillations falls into the low-frequency band [for pharmacological blockage study see Kromenacker et al., 2018]. Furthermore, tonic sympathetic activity levels can additionally indirectly influence the HF component measure (Chapleau and Sabharwal, 2011). That means that the HF component can be interpreted as reflecting cardiac parasympathetic activation only under specific circumstances. The low frequency (LF) component has previously been observed to increase in power through HRV BFB (Laborde et al., 2022a). This is due to the RSA frequency falling into the LF component during SPB, as described above. Since RSA operates within a different frequency band during spontaneous breathing, this component, like the HF component, does not assess vmHRV in a comparable manner. The RMSSD was therefore used as an indicator of vmHRV for each assessment. For each session, the RMSSD assessment at rest from the beginning of the session was used as baseline vagal activity (Laborde et al., 2017). The RMSSD was calculated in the BioSign^®^ Software “HRV-Scanner” following the formula:


$$\text{RMSSD}=\sqrt{\frac{1}{N}\times \sum_{i=1}^{N}{\left(RR_{i+1}-RR_{i}\right)}^{2}}.$$

#### 2.3.2. Biofeedback threshold assessment

An individual threshold was assessed for the BFB training. For the assessment, participants were asked to breathe as deeply as possible in a paced breathing rhythm at 0.1 Hz for 1 minute, as the ECG continued to record their heart rate following the protocol outlined in the documentation of the HVR-Scanner software (BioSign GmbH, 2023). At this rate, the breathing pace approaches the resonance frequency of most individuals and RSA is therefore strongly increased.

With this data, spectral analysis was performed through a Fast Fourier transform based on linearly interpolated R-R intervals in the HRV-Scanner software. In a patented algorithm by BioSign^®^ (Beise, 2010), two components are identified from the integral of this spectral analysis. The first one contains the integral around the peak of the variance distribution and the other one the leftover frequencies. The two components are then normed and adjusted. The ratio of the adjusted integral components indicates the relative contribution of RSA to the total variance of oscillations and is calculated to assess individual thresholds for the feedback in the biofeedback intervention. The precise algorithm for the calculation can be found in the registered patent by Beise (2010).

#### 2.3.3. BFB and NV

The biofeedback was presented as visual feedback in the form of a hot air balloon flying through a landscape. Participants saw the balloon rising when the current HRV component ratio, calculated as described in the segment above, rose toward and above their individual threshold based on the biofeedback threshold assessment (and fell when it dropped below the individual threshold). A detailed description of the task can be found in the software documentation (BioSign GmbH, 2023). Participants were provided with explanations on how the biofeedback system works, and that the rising balloon indicated synchronization of breath and heart rate. They were instructed to breathe in the induced frequency through their nose in a relaxed, natural way. Participants practiced the biofeedback for about a minute before the start of the exercise. If participants reported any tension or hyperventilation symptoms after the initial practice, the experimenter instructed them to practice shallower breathing and/or provided advice to comfortably slow down the breathing rate. If necessary, another one-minute practice session was conducted before the start of the exercise to ensure successful completion.

The ratio described above, which is used to quantify breath-to-heart-rate synchronization, was constantly recalculated, and updated to ensure swift visual feedback of the current RSA levels within the BioSign^®^ software. This algorithm was developed specifically to ensure a higher dynamic of online feedback.

A bar moving up and down indicated the slow-paced breathing rhythm at 0.1 Hz, which has been shown to maximize resonance between the breathing cycle and heart rate (Vaschillo et al., 2006). For the control condition, participants saw only a breathing bar on a black background, which dictated a breathing rhythm to imitate normoventilation at 0.25 Hz. For the HRV biofeedback, the biofeedback threshold assessment suggested a threshold of the ratio described above individually for each participant in order to achieve a comparable task difficulty.

The RMSSD was calculated for the ECG recordings during the intervention. The RMSSD baseline values were then subtracted from the RMSSD during the intervention for each session. This score indicates the gain (or decrease if the value is negative) in RMSSD during the intervention in comparison to the baseline, subsequently referred to as RMSSD_gain_.

### 2.4. Revised attention network test (ANT-R)

We used the ANT-R (Fan et al., 2009), based on the original attention network test (Fan et al., 2002), which is a reaction time paradigm combining the Eriksen flanker task (Eriksen and Eriksen, 1974) and the Posner cueing task (Posner, 1980).

In this task, participants respond to a target stimulus of a black horizontal arrow on a gray background. By pressing the correct button with either their left or right index finger, they are asked to decide in which direction the target arrow points.

The original ANT-R consists of 288 trials, in which the first and second runs (of the 144 trials each) are identical. The whole test lasts a total duration of 30 min. Prior studies showed high split-half reliability (Greene et al., 2008) in the Executive (*r* = 0.74) and Orienting network scores (*r* = 0.70). In order to achieve maximum effects and reduce strain on participants, only one of the two identical runs was completed by the participants per session.

The task was programmed and applied using the software Presentation^®^ (Neurobehavioral Systems, Inc., Berkeley, CA, www.neurobs.com) following the detailed description of Fan et al. (2009). The task was presented on a 24-inch screen, placed 80 cm away from the participants. Participants completed 6 practice trials with feedback and 32 practice trials without feedback after receiving written and visual instructions. A description of the results can be seen in Table 1.

### 2.5. Perceived stress scale

The perceived stress scale (PSS-10) is a short (10 items) scale assessing self-reported current stress experienced during the last 7 days (Cohen et al., 1983) on a 5-point Likert scale (0–4). The German version of the scale showed good internal consistency and construct validity in a large, representative sample (*N* > 2,400) (Klein et al., 2016).

### 2.6. Statistical analysis

All statistical analyzes were carried out in R Version 4.1.2. All linear mixed models were calculated using the lm4 package (v1.1–31) (Bates et al., 2015). Degrees of freedom and value of ps were calculated using the Satterthwaite’s degrees of freedom method in the lmerTest package (v3.1–31). RMSSD values as well as the reaction times were BoxCox transformed (Box and Cox, 1964) to account for skewness.

To test whether the BFB intervention successfully increased RMSSD compared to the control condition (Hypothesis 1), a linear mixed model was calculated for the intervention gain score (RMSSD_gain_). Participant intercepts were modeled as random effects. Four different models were fitted which included only the condition (BFB or NV) and then the successive addition of the RMSSD baseline, the interaction term of the condition and RMSSD baseline and the control variables (age, BMI, gender). The goodness of fit was then assessed through an ANOVA, checking for additional explained variance through a Likelihood Ratio Test from each model to the next complex one, yielding a value of p from the *χ*^2^-test [see proposition by Meteyard and Davies (2020)]. The Bayesian and Akaike Information Criteria (BIC and AIC) were also assessed but played a secondary role in the model selection. The best-fitting model was then used to test the hypothesis.

To test Hypotheses 2–4, a linear mixed model of reaction time (RT) was calculated. The mixed model has several advantages compared to traditional analyzes of the composite scores (Fan et al., 2009). First and foremost, the power of the statistic is increased, as a mixed model allows the inclusion of each trial into the analysis instead of just one data point per session (resulting in up to a 96-fold increase in data points). Secondly, there is a loss of information and distortion of the effects when analyzing the composite scores. The composite scores are calculated by subtracting the average reaction times of correctly answered trials of different conditions from each other (e.g., mean RT of trials with invalid cues – mean RT of trials with valid cues). In the ANT-R, each trial consists of a cue and flanker condition. If the participant answers 100% of the trials correctly, the mean RT_valid cue_ and RT_invalid cue_ then include 50% of trials with congruent and incongruent flankers. However, accuracy is not the same for all conditions. Each participant has an individual loss in accuracy depending on the condition. Typically, there will be more wrong answers in the invalid cue, incongruent flanker condition than in the valid cue, incongruent flanker condition. This leads the mean RT_invalid cue_ to include more congruent flanker trials than the RT_valid cue_, which are responded to faster and mask part of the orienting effect. In the composite scores, it is therefore not possible to differentiate between the individual contributions of the conditions. A mixed model includes each trial, which enables accurate identification of the contribution of the cue and flanker to the reaction time.

To maintain parsimonious models and reduce convergence issues, the random effect structure was kept simple and included only intercepts in participants to account for individual differences in average reaction time and allow for subject-wise clustering of the data points. In an iterative process similar to the one described above, predictors were consecutively added to the fixed effects, with each step of complexity being tested for improvement of fit through Likelihood Ratio Testing to the next simpler model. Predictors were maintained for the next step if they improved the model. Predictors included in the model were flanker, cue, RMSSD baseline, condition, PSS, age, gender, BMI, RMSSD * cue interaction, cue * condition interaction and cue * condition * PSS interaction.

The simplest model included only the factors of flanker (2 levels: congruent/incongruent) and cue (2 levels: valid/invalid), which equal the Executive and Orienting network contributions to the reaction times. The relevant factors were dummy coded so that positive slopes would indicate the expected effects (i.e., slower responses in incongruent and invalid trials) to facilitate interpretation. Trials with double or no cues were not included in the analysis because they are used to assess the Alerting Network, which was not the focus of our hypotheses in this study. Trials assessing the Orienting Network (congruent/incongruent cues) and the Alerting Network (no/double cues) are mutually exclusive and, as such, cannot be simultaneously modeled. This results in up to 108 trials (comprising 72 valid cues and 36 invalid cues) per participant per condition, dependent on individual accuracy levels. The hypotheses were accepted when the final model included the RMSSD * cue interaction (Hypothesis 2), the cue * condition interaction (Hypothesis 3) and the cue * condition * PSS interaction (Hypothesis 4), with each interaction being a significant predictor of reaction time and the interaction effect pointing in the expected direction.

## 3. Results

The BoxCox transform showed **λ**-values between 0 and − 0.5 for RMSSD baseline data and RMSSD intervention data. This indicates that either a log(RMSSD) (for 0) or a − 1/sqrt(RMSSD) (for −0.5) transform is appropriate to correct for skewness of the data. To maintain consistency between the data and enable comparability with other HRV research (Sørensen et al., 2019), a logarithmic transform was applied. Visual inspection of the distribution of the log-transformed RMSSD data revealed approximately normal distributions.

Similarly, BoxCox analysis showed a positive skew of the raw reaction time data. Approximate normal distribution was achieved through log transform.

All participants completed the ANT-R with at least 80% accuracy in the main block and 96% accuracy on average. T-tests for dependent samples showed no significant difference in accuracy between conditions (*t* = 0.40, *p* = 0.69) and a slight improvement over sessions (mean_accuracy t1_ = 0.944, mean_accuracy t2_ = 0.955, *t* = 2.67, *p* < 0.05).

### 3.1. Hypothesis 1

The first hypothesis was that the HRV BFB intervention yields a higher RMSSD than the normoventilation condition. The first model predicting RMSSD_gain_ (from baseline) during the intervention included only the condition as a fixed effect. Adding the HRV baseline as a predictor improved the model. This is reflected in both the highly significant Chi-squared value for the additional explained variance and the lower AIC and BIC values. The interaction term between RMSSD baseline and the condition did not significantly improve the fit, as seen in all three indicators. Including sociodemographic parameters also did not improve the model fit.

The fit that predicts RMSSD_gain_ (from the intervention) through the condition and RMSSD baseline was therefore chosen as the model with the best fit and used to test H1 (see Table 2 for results). The condition was a significant predictor of RMSSD_gain_ [(RMSSD during intervention) – (RMSSD baseline)] in this model (*p* < 0.001).

**Table 2:** Results of the mixed model with the best fit predicting RMSSD_gain_ during intervention.

Predictors	Standardized RMSSD_gain_					
	*β*	std. Error	CI	*t* value	*p*	*df*
Intercept	−0.75	0.08	−0.91–−0.60	−9.77	**<0.001**	111
RMSSD	−0.28	0.06	−0.39–−0.17	−4.94	**<0.001**	69
condition	1.51	0.11	1.30–1.72	14.34	**<0.001**	56
Random effects						
σ^2^	0.31					
τ_00vpn_	0.02					
ICC	0.07					
N_vpn_	56					
Observations	112					
Marginal R^2^ / Conditional R^2^	0.666 / 0.689					

RMSSD_gain_ is calculated through log(RMSSD during the intervention)-log(RMSSD baseline). Participant intercepts are modeled as random factors. Values reported are standardized regression weights (*β*). RMSSD, root mean square of successive differences. Bold *p*-values indicate significant effects.

**Figure 2:**
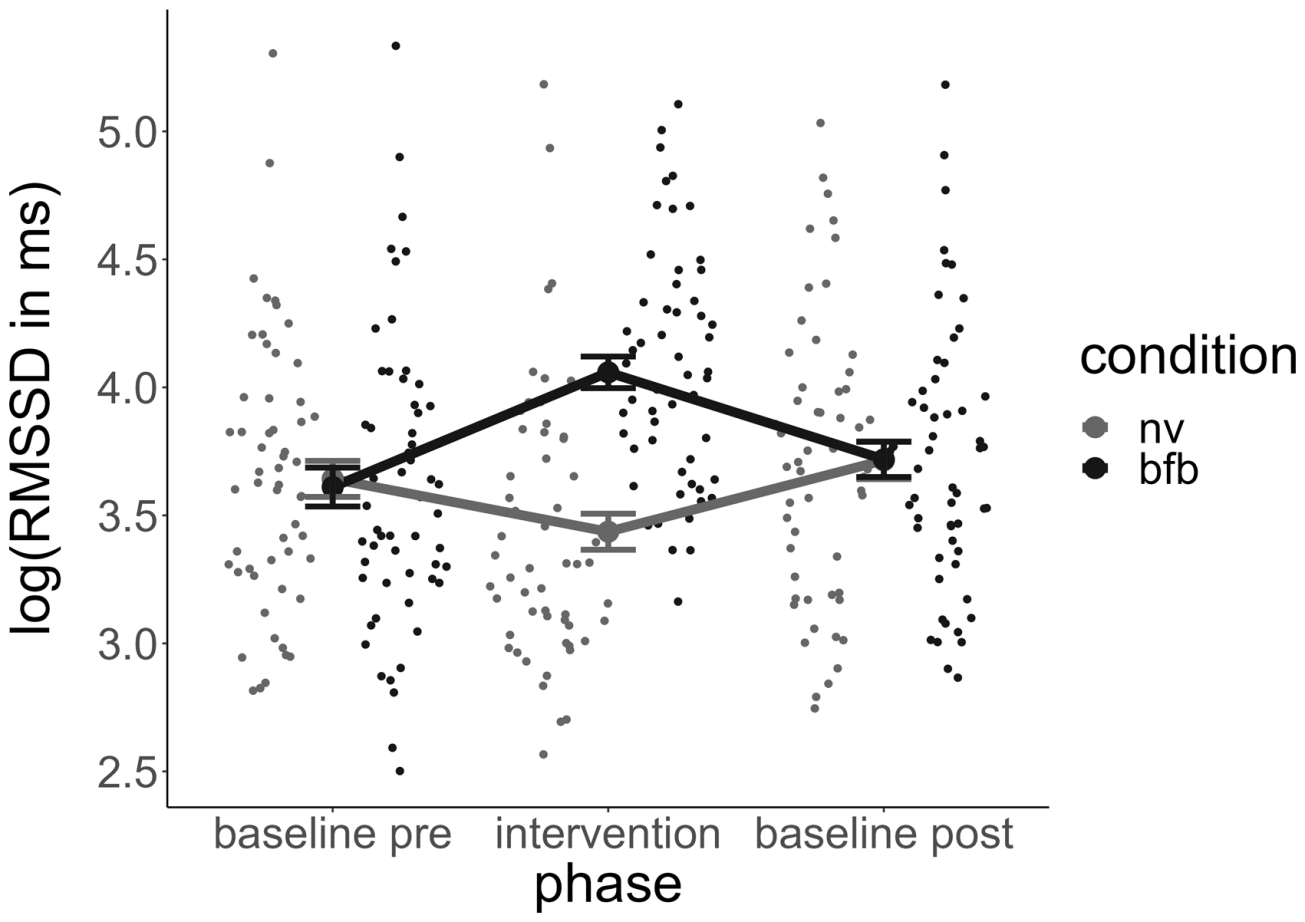
Course of RMSSD across experimental phases by condition. Error bars indicate standard errors. RMSSD, root mean square of successive differences; BFB, biofeedback; NV, normoventilation.

Figure 2 indicates that the direction of the effect was in the expected direction. RMSSD_gain_ through the BFB condition was higher than through the control condition of paced breathing at a normoventilation rate. The standardized regression coefficient of the condition in the linear mixed model was *β* = 1.5, which implies that during the BFB, RMSSD had increased 1.5 standard deviations more than during the NV from the baseline on average. T-tests for dependent samples verified that in the BFB condition the RMSSD during the intervention was significantly higher than the RMSSD baseline (*t* = 4.57, *p* < 0.001), whereas during the control condition the RMSSD was slightly lowered (*t* = −2.07, *p* < 0.05).

A negative β of the RMSSD baseline as a predictor indicates that individuals with a higher RMSSD baseline respond to the BFB intervention to a lower degree.

### 3.2. Hypotheses 2–4

The model with the best fit to the present reaction time data is the following:

RT ~ condition:cue:PSS + condition + PSS + cue * RMSSD_baseline_ + flanker + (1 | participant).

Each fixed effect slope and interaction included in this model is a significant predictor. For standardized regression weights and individual R^2^ contributions of the model, see Table 3.

**Table 3:** Results of the model with the best fit for reaction times.

Predictors	Standardized reaction time					
	*β*	std. Error	CI	*t* value	*p*	*df*
Intercept	−0.76	0.05	−0.86–−0.65	−13.87	**<0.001**	60
PSS	0.09	0.05	−0.02–0.19	1.60	0.109	56
condition	−0.04	0.01	−0.06–−0.01	−2.39	**0.017**	10,010
cue	0.76	0.02	0.73–0.79	44.52	**<0.001**	9,991
RMSSD	−0.07	0.02	−0.12–−0.03	−3.13	**0.002**	1979
Flanker	0.95	0.01	0.92–0.98	64.74	**<0.001**	9,991
cue × RMSSD	−0.07	0.02	−0.11–−0.04	−4.22	**<0.001**	9,991
(condition × cue) × PSS	−0.07	0.02	−0.12–−0.03	−3.19	**0.001**	10,020
Random effects						
σ^2^	0.53					
τ_00vpn_	0.16					
ICC	0.23					
N_vpn_	56					
Observations	10,047					
Marginal *R*^2^ / Conditional *R*^2^	0.329 / 0.482					

Participant intercepts are modeled as random factors. *β*, standardized regression weight; RMSSD, root mean square of successive differences; PSS, perceived stress scale. Bold *p*-values indicate significant effects.

#### 3.2.1. Hypothesis 2

**Figure 3:**
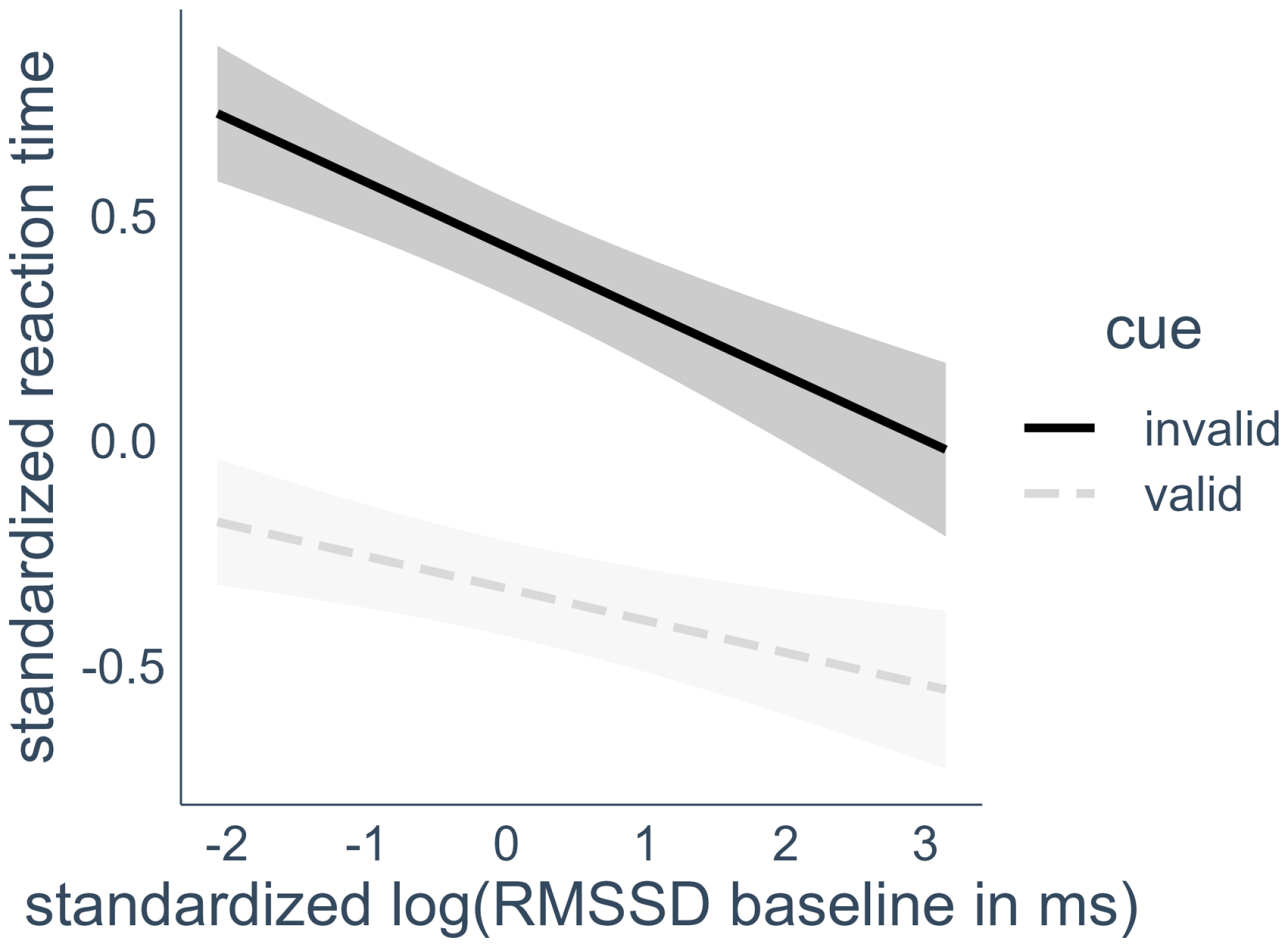
Interaction effect of heart rate variability and cue condition on reaction time. RMSSD, root mean square of successive differences; valid cues, spatial cues in the same location that target appears in later; invalid cues, spatial cues in a different location than the target. Highlighted areas indicate 95% confidence intervals.

H2 stated that a higher RMSSD baseline, measured at rest, would predict better performance (lower scores) in the Orienting Network score of the ANT-R over both HRV biofeedback and control conditions. The cue * RMSSD baseline interaction confirmed the hypothesized association between the Orienting Network and RMSSD. Visual inspection of the interaction effect showed that the effect is in line with the hypothesized direction (see Figure 3). This was confirmed in post-hoc testing. This was confirmed in post-hoc testing. We compared the estimated marginal means of the linear trends that compared the slope of RMSSD predicting RT in valid and invalid cue trials using the emmeans package (v1.8.8). The marginal slope of the invalid trials was significantly steeper (−0.14) than the slope of the valid trials (−0.07), *d* = 0.07, *z_ratio_* = 4.22, *p* < 0.001, leading to less difference between the slopes the higher the RMSSD.

Participants responded faster in trials with invalid cues the higher their RMSSD baseline was. This effect was also visible in a bivariate Pearson correlation of −0.22 (p < 0.05) between RMSSD baseline and the Orienting Score (calculated RT_invalid cue_ - RT_valid cue_) when including only control condition data points in the calculation. As expected, higher RMSSD at rest was associated with better Orienting Network performance.

#### 3.2.2. Hypothesis 3

We hypothesized that the Orienting Network scores of the ANT-R performance would be better after an HRV biofeedback intervention compared to the control condition. The cue * condition interaction neither appeared in the final model nor proved to be a significant predictor in any iteration. Contrary to Hypothesis 3, HRV BFB did not significantly improve the Orienting Network performance.

#### 3.2.3. Hypothesis 4

We also postulated that the current stress level moderates the effect of HRV BFB on the Orienting Network performance of the ANT-R.

**Figure 4:**
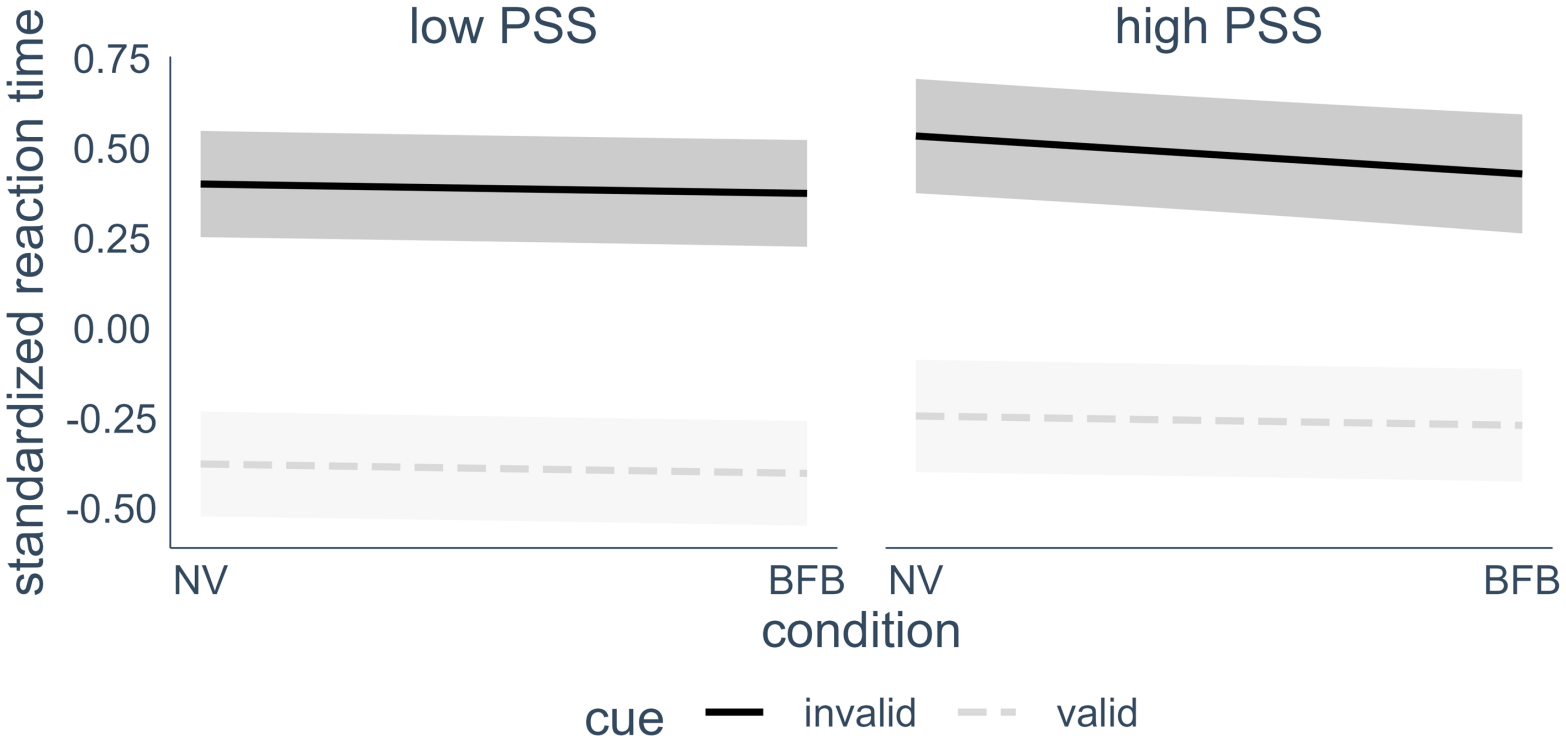
Interaction effect of current perceived stress (median split), cue condition and intervention on reaction time. For visualization purposes, a median split was conducted that separated the participants into a low stress (*n* = 30) and a high stress (*n* = 26) group. In the analyzes however, the PSS score was treated as a continuous variable. Highlighted areas indicate 95% confidence intervals. PSS, perceived stress scale; NV, normoventilation; BFB, biofeedback; valid cues, spatial cues in the same location that target appears in later; invalid cues, spatial cues in a different location than the target.

The 3-way interaction of self-reported stress, condition (normoventilation or biofeedback) and cue (valid or invalid spatial cue) appeared as a significant predictor of reaction time in the final model (p < 0.05). A visualization of the effect (see Figure 4) revealed that the biofeedback had a differential effect on the Orienting Network reaction times depending on the stress level of the individuals. In line with Hypothesis 4, there was a beneficial effect of the BFB for participants who reported high levels of stress, compared to less stressed individuals. Although there was no overall effect of HRV BFB on Orienting Network performance, in individuals with higher stress levels reaction times were lower in invalid spatial cue trials after the BFB condition compared to the normoventilation condition. The effect was quite small, however, with about 0.5% unique additional variance explained when adding this effect. To confirm the hypothesis, we conducted post-hoc Tukey testing. In order to facilitate the interpretation of the post-hoc testing, we conducted a median split with the PSS values and then performed a Tukey test on the three-way interaction, including PSS as a factor. The Tukey test confirmed that the interaction was driven by a significant difference between the NV and the BFB condition only in invalid cue trials in the high PSS group, *d* = 0.11, *z_ratio_* = 2.81, *p* < 0.01. None of the other NV vs. BFB comparisons were significant.

## 4. Discussion

In the present study, we explored the acute effects of HRV BFB on attentional control. The HRV BFB had the expected effect of increasing the vmHRV during the intervention compared to the paced normal ventilation condition and the baseline. The data also revealed the expected association of a higher vmHRV baseline with better (lower) scores in the ANT-R Orienting Score. The Orienting Score was not significantly improved by the biofeedback intervention over all participants. However, an interaction effect of current self-reported stress levels and the condition showed that individuals with high stress performed slightly better in the Orienting Score after the biofeedback condition compared to the control condition.

### 4.1. Mechanisms of HRV biofeedback

The improved vmHRV during the HRV BFB condition implies that the BFB fulfilled its intended function of increasing the vmHRV at least temporarily during the intervention. As can be observed in Figure 2, the post-HRV baseline, assessed approximately 15–25 min after the intervention, did not differ between the biofeedback and control conditions. The effects of vmHRV improvement through the BFB therefore seem to have been rather short-lived.

This might be due either to the actual transience of the effects or to the circumstance that during this time participants engaged in a cognitive task. Active engagement usually leads to a reactive drop in vmHRV (Laborde et al., 2017), and therefore might have counteracted a possible vmHRV improvement over a longer time frame. However, previous research suggests that the increased vmHRV during slow-paced breathing returns to baseline shortly after the intervention, even without a reactive drop due to task engagement (You et al., 2022). Taken together, these findings suggest relatively short-term effects on improving vmHRV through a single session of HRV BFB.

The proposed mechanisms of feedback loops and temporal coherence between heart rate and breathing cycle (Lehrer and Gevirtz, 2014; Sevoz-Couche and Laborde, 2022) are supported by the present data. In line with the authors’ claim that the heart rate, breathing and blood pressure oscillations resonate at a local maximum frequency of 0.1 Hz, our data showed a strong increase in vmHRV when participants were asked to breathe at a rate of 0.1 Hz but not when breathing at a rate of 0.25 Hz.

Not all participants were able to profit from the BFB. Some did not show an improvement in vmHRV. This is likely due to the very short intervention with only minimal coaching. Learning to adjust physiological processes in the body is no easy task and can therefore not necessarily be expected to be learned in 5 min but may require more intensive training. Excluding non- or low responders from the analysis, however, did not change the results. The association of HRV baseline and the HRV gain through biofeedback must also be noted. Participants who had a higher resting vmHRV were profiting less from the biofeedback, indicating potential ceiling effects.

The fact that the control condition, which also induced paced breathing, did not lead to an increase in vmHRV speaks against the contribution of simple concentrative effects of HRV BFB on cardiac parasympathetic activation. As a matter of fact, the control condition even had a slightly detrimental effect on the vmHRV. This finding is in line with previous research which has extensively shown that increases in vmHRV during paced breathing are only found in low frequencies between 4 and 7 cycles per minute (e.g., Song and Lehrer, 2003). The literature has also showed the superiority of HRV BFB over concentrative/meditative practices, such as progressive muscle relaxation, in improving vmHRV (Huang et al., 2018; Lin et al., 2020).

### 4.2. Resting vmHRV and attentional control

The associations between the Orienting Score of the ANT-R and the vmHRV baseline at rest (RMSSD) (Ramírez et al., 2015; Quintana et al., 2017; Sørensen et al., 2019) were replicated in this study. Consistent with prior attentional control research (Zahn et al., 2016; Holzman and Bridgett, 2017), a higher vmHRV at rest was associated with better top-down goal-directed attentional control as indicated by lower Orienting Scores.

Within the ANT-R Orienting paradigm, valid spatial cues outweigh invalid ones (3:1). This leads to a positive expectation of the appearance of the stimuli in the indicated location and the conditions’ orientation toward the cued location, leading to consistently faster reaction times in trials with valid vs. invalid cueing. The activity of the amygdala, among others, is related to the conditioned allocation of spatial attention (Vuilleumier, 2009). The theory of neurovisceral integration (Thayer and Lane, 2009) proposes that non-ideal conditioned behavior patterns such as this conditioned attention allocation can be attuned through functional inhibitory connectivity from vmPFC to the amygdala, which can therefore enable efficient reorientation after an invalid cue. According to the model of neurovisceral integration (Thayer et al., 2009), this functional connectivity is reflected in vmHRV measures. The data found in this study – an association between the physiological marker of CAN capacity (HRV) and the efficiency in spatial attention reallocation – offers further evidence for this theoretical framework.

Overall, the BFB intervention did not improve attentional control. After the HRV BFB intervention, participants did not achieve significantly better values in the Orienting Score, which contradicts our expectations based on prior findings of associations between the constructs. The present findings speak against cognitive effects from simply “activating” the CAN by means of afferent vagal activation through the BFB. It is possible that effects can only be observed after the strengthening of the connectivity and activity of the CAN through long-term BFB training. The results also do not support the findings of Prinsloo et al. (2013a), who found acute improvement on a cognitive task after BFB, although their study was severely underpowered with a sample of only seven individuals in the biofeedback and nine individuals in the control condition. A major difference between the two studies as well as from the other studies finding positive effects (Sherlin et al., 2010; Prinsloo et al., 2011; Laborde et al., 2019) is that the current study was conducted with a healthy sample, whereas the previous studies investigated highly stressed individuals or individuals who had just been exposed to a stressor.

### 4.3. HRV biofeedback, attentional control and stress

We also included current stress, measured as self-reports using the Perceived Stress Scale, in the analysis. The interaction term of condition and stress was a significant predictor of attentional control. This revealed that the BFB had different effects in different subgroups. The more stressed the participants were, the more they could profit from the biofeedback intervention regarding their attentional control capacity. Similarly, a previous study found that a single-session HRV BFB reduced anxiety only in individuals with high baseline anxiety in a sample of musicians (Wells et al., 2012).

The most probable mechanistic pathway, which explains differential effects dependent on stress levels, is implied by attentional control theory (Eysenck et al., 2007). This theory states that deficits in attentional control are elicited by stress and anxiety. Stress is related to enhanced worrying, which in turn withdraws attentional resources from current tasks. Additionally, a state of anxiety induces preferential stimulus-driven, bottom-up attention direction. HRV BFB has been shown to momentarily decrease anxiety and increase calmness in stressed students (Meier and Welch, 2016), which might directly reduce these detrimental effects only in participants who were affected in the first place.

The current acute effects of HRV BFB on attentional control in stressed participants would also speak for the feasibility of immediate activation of the central autonomic network in individuals whose CAN either shows a lower functional capacity or less frequent activation. Mechanisms through which this direct enhancement of cognitive functioning through HRV BFB can occur include the afferent pathway (Lehrer and Gevirtz, 2014).

Another possible mechanism for the acute cognitive effects is the direct mechanical pathway from the olfactory bulb to cortical areas. The sensory registration of airflow through the nose produces slow cortical potentials (SCPs) in the same frequency as the rhythmic air flow (Zaccaro et al., 2022). These SCPs have enhancing effects on self-regulation and voluntary attention allocation, which is why SCP biofeedback is an effective treatment for attention deficit disorders (Zaccaro et al., 2018).

It must be noted, however, that the differential effect of HRV BFB on attentional control depending on stress levels is quite small (only 0.15% of the variance was explained across all reaction time trials). In fact, the effect is only visible when variance due to flanker condition has been removed from the raw reaction time data. The raw average composite scores do not show the interaction effect. Whether or not the effect size amounts to a noticeable practical effect in high acute or chronic stress would have to be further investigated with specific target populations.

### 4.4. Limitations and prospects for future research

A limitation comes from conducting this study during the onset and course of the Covid-19 pandemic. The conditions under which the study was conducted were especially strict (face mask, interpersonal distance, regular disinfection, etc.), which might have decreased the potential for the relaxing effects of the HRV BFB.

Another limitation is the length of the biofeedback intervention. Participants only practiced the biofeedback for a total of about 6 min. Whether or not this is enough to induce effects that last for at least 15 min (the duration of the ANT-R) is not clear. The previously conducted studies investigating acute effects on cognitive functions each employed slow-paced breathing or HRV BFB interventions with time durations between 10 and 17 min (Sherlin et al., 2010; Prinsloo et al., 2011; Hoffmann et al., 2019; Laborde et al., 2019, 2022b). This may shed light on why there was no main effect found in this study.

Additionally, the control condition of induced paced breathing to simulate normoventilation had a slightly detrimental effect on vmHRV, which might have subsequently altered the reaction time performance. While it was useful to specify the mechanisms through which HRV BFB is able to unfold its effect, it might have altered the ANT-R scores in an unexpected way, which thus might have covered up the effects. A clear implication for future research would be to focus on a sample of individuals with a compromised vmHRV, such as persons under acute stress (Sherlin et al., 2010; Prinsloo et al., 2011; Hoffmann et al., 2019; Laborde et al., 2019, 2022b), who are likely able to profit more from the BFB intervention (Mayer et al., 2013) and therefore have a higher chance of also profiting from acute effects on attentional control. HRV BFB did not increase resting vmHRV in all individuals, also among those with a below average vmHRV. Future studies should investigate what differentiates individuals who can profit from the BFB from those who cannot. Instructions and coaching might also have to be adjusted in order to ensure benefits for all participants. An interesting prospect would also be to test the effects of long-term HRV BFB training on the same cognitive parameters to compare the effects of functional training and strengthening of the CAN capacity to the acute activation. Future research might also look more closely at the duration of the acute effects of HRV BFB. The results of this study showed that 15–20 min after a 5-min intervention, vmHRV was already back at baseline. This might be due to a rebound reaction from the reaction time task (Mezzacappa et al., 2001; Siepmann et al., 2008) or the actual transience of the effects.

To further investigate the specificity and the mechanisms by which HRV BFB affects stressed individuals, conducting a comparative study between HRV BFB and another relaxation technique, such as progressive muscle relaxation, would be essential. Additionally, a study design that assesses attentional control before and after the intervention, rather than on two separate days as seen in other studies examining the acute effects of HRV BFB (e.g., Prinsloo et al., 2011), could help minimize confounding variables and might be better suited to detect effects.

## 5. Conclusion

In conclusion, we found differential acute effects of a single-session 5-min HRV BFB intervention on attentional control. Highly stressed individuals profited from the intervention, whereas less stressed individuals did not and might even show adverse effects. The effect was quite small, however, which warrants further research into the nature of this effect.

## Data availability statement

The raw data supporting the conclusions of this article will be made available by the authors, without undue reservation.

## Ethics statement

The studies involving humans were approved by the ethics committee of the University of Potsdam (no 15/2021). The studies were conducted in accordance with the local legislation and institutional requirements. The participants provided their written informed consent to participate in this study.

## Author contributions

BB: Data curation, Formal analysis, Investigation, Methodology, Project administration, Software, Supervision, Validation, Visualization, Writing – original draft. MW: Resources, Supervision, Writing – review & editing. JW: Conceptualization, Methodology, Project administration, Resources, Supervision, Writing – review & editing.

## References

[ref1] Bates D. Mächler M. Bolker B. Walker S. (2015). Fitting linear mixed-effects models using lme4. J. Stat. Soft. 67:67. doi: 10.18637/jss.v067.i01

[ref2] Beise R. D. (2010), Inventor; Biosign Medical UGhb, assignee. Method for evaluating heart rate variability. US7860560B2.

[ref3] Berntson G. G. Cacioppo J. T. Quigley K. S. (1993). Respiratory sinus arrhythmia: autonomic origins, physiological mechanisms, and psychophysiological implications. Psychophysiology 30, 183–196. doi: 10.1111/j.1469-8986.1993.tb01731.x, PMID: 8434081

[ref4] BioSign GmbH (2023). Documentation for HRV-scanner. Available at: https://www.biosign.de/download_HRVScanner/HRV-Scanner%20Dokumentation%20Part%201%20EN.pdf

[ref5] Box G. E. P. Cox D. R. (1964). An analysis of transformations. J. R Stat. Soc. Series B Stat. Methodol. 26, 211–243. doi: 10.1111/j.2517-6161.1964.tb00553.x

[ref6] Chapleau M. W. Sabharwal R. (2011). Methods of assessing vagus nerve activity and reflexes. Heart Fail. Rev. 16, 109–127. doi: 10.1007/s10741-010-9174-6, PMID: 20577901 PMC4322860

[ref7] Cohen S. Kamarck T. Mermelstein R. (1983). A global measure of perceived stress. J. Health Soc. Behav. 24, 385–396. doi: 10.2307/2136404 6668417

[ref8] Da Silva V. P. Oliveira N. A. d. Silveira H. Mello R. G. T. Deslandes A. C. (2015). Heart rate variability indexes as a marker of chronic adaptation in athletes: a systematic review. Ann. Noninv. Electrocard. 20, 108–118. doi: 10.1111/anec.12237, PMID: 25424360 PMC6931675

[ref9] Eckberg D. L. (1983). Human sinus arrhythmia as an index of vagal cardiac outflow. J. Appl. Physiol. Respir. Environ. Exerc. Physiol. 54, 961–966. doi: 10.1152/jappl.1983.54.4.961, PMID: 6853303

[ref10] Eriksen B. A. Eriksen C. W. (1974). Effects of noise letters upon the identification of a target letter in a nonsearch task. Percept. Psychophys. 16, 143–149. doi: 10.3758/bf03203267

[ref11] Eysenck M. W. Derakshan N. Santos R. Calvo M. G. (2007). Anxiety and cognitive performance: attentional control theory. Emotion 7, 336–353. doi: 10.1037/1528-3542.7.2.336 17516812

[ref12] Fan J. Gu X. Guise K. G. Liu X. Fossella J. Wang H.. (2009). Testing the behavioral interaction and integration of attentional networks. Brain Cogn. 70, 209–220. doi: 10.1016/j.bandc.2009.02.002, PMID: 19269079 PMC2674119

[ref13] Fan J. McCandliss B. D. Sommer T. Raz A. Posner M. I. (2002). Testing the efficiency and independence of attentional networks. J. Cogn. Neurosci. 14, 340–347. doi: 10.1162/089892902317361886, PMID: 11970796

[ref14] Faul F. Erdfelder E. Lang A.-G. Buchner A. (2007). G*power 3: a flexible statistical power analysis program for the social, behavioral, and biomedical sciences. Behav. Res. Methods 39, 175–191. doi: 10.3758/BF03193146, PMID: 17695343

[ref15] Gerritsen R. J. S. Band G. P. H. (2018). Breath of life: the respiratory vagal stimulation model of contemplative activity. Front. Hum. Neurosci. 12:397. doi: 10.3389/fnhum.2018.00397, PMID: 30356789 PMC6189422

[ref16] Goessl V. C. Curtiss J. E. Hofmann S. G. (2017). The effect of heart rate variability biofeedback training on stress and anxiety: a meta-analysis. Psychol. Med. 47, 2578–2586. doi: 10.1017/S0033291717001003, PMID: 28478782

[ref17] Greene D. J. Barnea A. Herzberg K. Rassis A. Neta M. Raz A.. (2008). Measuring attention in the hemispheres: the lateralized attention network test (LANT). Brain Cogn. 66, 21–31. doi: 10.1016/j.bandc.2007.05.003, PMID: 17590491 PMC4283820

[ref18] Grossman P. Taylor E. W. (2007). Toward understanding respiratory sinus arrhythmia: relations to cardiac vagal tone, evolution and biobehavioral functions. Biol. Psychol. 74, 263–285. doi: 10.1016/j.biopsycho.2005.11.014, PMID: 17081672

[ref19] Henderson L. A. Richard C. A. Macey P. M. Runquist M. L. Yu P. L. Galons J.-P.. (2004). Functional magnetic resonance signal changes in neural structures to baroreceptor reflex activation. J. Appl. Physiol. 96, 693–703. doi: 10.1152/japplphysiol.00852.2003 14565965

[ref20] Hoffmann S. Jendreizik L. T. Ettinger U. Laborde S. (2019). Keeping the pace: the effect of slow-paced breathing on error monitoring. Int. J. Psychophysiol. 146, 217–224. doi: 10.1016/j.ijpsycho.2019.10.001, PMID: 31669325

[ref21] Holzman J. B. Bridgett D. J. (2017). Heart rate variability indices as bio-markers of top-down self-regulatory mechanisms: a meta-analytic review. Neurosci. Biobehav. Rev. 74, 233–255. doi: 10.1016/j.neubiorev.2016.12.032, PMID: 28057463

[ref22] Huang C. Gevirtz R. N. Onton J. Criado J. R. (2018). Investigation of vagal afferent functioning using the heartbeat event related potential. Int. J. Psychophysiol. 131, 113–123. doi: 10.1016/j.ijpsycho.2017.06.007, PMID: 28679109

[ref23] Hunter J. F. Olah M. S. Williams A. L. Parks A. C. Pressman S. D. (2019). Effect of brief biofeedback via a smartphone app on stress recovery: randomized experimental study. JMIR Serious Games 7:e15974. doi: 10.2196/15974, PMID: 31769761 PMC6904898

[ref24] Karemaker J. M. (2022). The multibranched nerve: vagal function beyond heart rate variability. Biol. Psychol. 172:108378. doi: 10.1016/j.biopsycho.2022.108378, PMID: 35688294

[ref25] Klein E. M. Brähler E. Dreier M. Reinecke L. Müller K. W. Schmutzer G.. (2016). The 30 German version of the Perceived Stress Scale - psychometric characteristics in a 31 representative German community sample. BMC Psychiatry 16:159. doi: 10.1186/s12888-016-0875-9, PMID: 27216151 PMC4877813

[ref26] Koenig J. Jarczok M. N. Kuhn W. Morsch K. Schäfer A. Hillecke T. K.. (2013). Impact of caffeine on heart rate variability: a systematic review. J. Caffeine Res. 3, 22–37. doi: 10.1089/jcr.2013.0009

[ref27] Kollai M. Mizsei G. (1990). Respiratory sinus arrhythmia is a limited measure of cardiac parasympathetic control in man. J. Physiol. 424, 329–342. doi: 10.1113/jphysiol.1990.sp018070, PMID: 2391653 PMC1189816

[ref28] Koskinen P. Virolainen J. Kupari M. (1994). Acute alcohol intake decreases short-term heart rate variability in healthy subjects. Clin. Sci. (Lond.) 87, 225–230. doi: 10.1042/cs0870225, PMID: 7924168

[ref29] Kromenacker B. W. Sanova A. A. Marcus F. I. Allen J. J. B. Lane R. D. (2018). Vagal mediation of low-frequency heart rate variability during slow yogic breathing. Psychosom. Med. 80, 581–587. doi: 10.1097/PSY.0000000000000603, PMID: 29771730

[ref30] Laborde S. Allen M. S. Borges U. Dosseville F. Hosang T. J. Iskra M.. (2022a). Effects of voluntary slow breathing on heart rate and heart rate variability: a systematic review and a meta-analysis. Neurosci. Biobehav. Rev. 138:104711. doi: 10.1016/j.neubiorev.2022.104711, PMID: 35623448

[ref31] Laborde S. Allen M. S. Borges U. Hosang T. J. Furley P. Mosley E.. (2022b). The influence of slow-paced breathing on executive function. J. Psychophysiol. 36, 13–27. doi: 10.1027/0269-8803/a000279

[ref32] Laborde S. Allen M. S. Borges U. Iskra M. Zammit N. You M.. (2022c). Psychophysiological effects of slow-paced breathing at six cycles per minute with or without heart rate variability biofeedback. Psychophysiology 59:e13952. doi: 10.1111/psyp.13952 34633670

[ref33] Laborde S. Lentes T. Hosang T. J. Borges U. Mosley E. Dosseville F. (2019). Influence of slow-paced breathing on inhibition after physical exertion. Front. Psychol. 10:1923. doi: 10.3389/fpsyg.2019.01923, PMID: 31507488 PMC6715106

[ref34] Laborde S. Mosley E. Mertgen A. (2018). Vagal tank theory: the three Rs of cardiac vagal control functioning - resting, reactivity, and recovery. Front. Neurosci. 12:458. doi: 10.3389/fnins.2018.00458, PMID: 30042653 PMC6048243

[ref35] Laborde S. Mosley E. Thayer J. F. (2017). Heart rate variability and cardiac vagal tone in psychophysiological research - recommendations for experiment planning, data analysis, and data reporting. Front. Psychol. 8:213. doi: 10.3389/fpsyg.2017.00213, PMID: 28265249 PMC5316555

[ref1002] Laborde S. Ackermann S. Borges U. D’Agostini M. Giraudier M. Iskra M.. (2023). Leveraging vagally-mediated heart rate variability as an actionable non-invasive biomarker for self-regulation: Assessment, Intervention, Evaluation. Policy Ins. Behav. Brain Sci. 10, 212–220. doi: 10.1177/23727322231196789, PMID: 32385728

[ref36] Lehrer P. M. Gevirtz R. (2014). Heart rate variability biofeedback: how and why does it work? Front. Psychol. 5:756. doi: 10.3389/fpsyg.2014.00756, PMID: 25101026 PMC4104929

[ref37] Lehrer P. M. Kaur K. Sharma A. Shah K. Huseby R. Bhavsar J.. (2020). Heart rate variability biofeedback improves emotional and physical health and performance: a systematic review and Meta analysis. Appl. Psychophysiol. Biofeedback 45, 109–129. doi: 10.1007/s10484-020-09466-z, PMID: 32385728

[ref38] Lehrer P. M. Vaschillo E. Vaschillo B. Lu S.-E. Eckberg D. L. Edelberg R.. (2003). Heart rate variability biofeedback increases baroreflex gain and peak expiratory flow. Psychosom. Med. 65, 796–805. doi: 10.1097/01.psy.0000089200.81962.19, PMID: 14508023

[ref39] Lehrer P. M. Vaschillo B. Zucker T. Graves J. Katsamanis M. Aviles M.. (2013). Protocol for heart rate variability biofeedback training. Biofeedback 41, 98–109. doi: 10.5298/1081-5937-41.3.08

[ref40] Lin I.-M. Wang S.-Y. Fan S.-Y. Peper E. Chen S.-P. Huang C.-Y. (2020). A single session of heart rate variability biofeedback produced greater increases in heart rate variability than autogenic training. Appl. Psychophysiol. Biofeedback 45, 343–350. doi: 10.1007/s10484-020-09483-y, PMID: 32767160

[ref41] Mayer K. Wyckoff S. N. Strehl U. (2013). One size fits all? Slow cortical potentials neurofeedback: a review. J. Atten. Disord. 17, 393–409. doi: 10.1177/1087054712468053, PMID: 23264371

[ref42] Meier N. F. Welch A. S. (2016). Walking versus biofeedback: a comparison of acute interventions for stressed students. Anxiety Stress Coping 29, 463–478. doi: 10.1080/10615806.2015.1085514, PMID: 26340374

[ref43] Meteyard L. Davies R. A. (2020). Best practice guidance for linear mixed-effects models in psychological science. J. Mem. Lang. 112:104092. doi: 10.1016/j.jml.2020.104092

[ref44] Mezzacappa E. S. P. Kelsey R. M. Katkin E. S. Sloan R. P. (2001). Vagal rebound and recovery from psychological stress. Psychosom. Med. 63, 650–657. doi: 10.1097/00006842-200107000-00018, PMID: 11485119

[ref45] Molfino A. Fiorentini A. Tubani L. Martuscelli M. Rossi Fanelli F. Laviano A. (2009). Body mass index is related to autonomic nervous system activity as measured by heart rate variability. Eur. J. Clin. Nutr. 63, 1263–1265. doi: 10.1038/ejcn.2009.35 19471292

[ref46] Noble D. J. Hochman S. (2019). Hypothesis: pulmonary afferent activity patterns during slow, deep breathing contribute to the neural induction of physiological relaxation. Front. Physiol. 10:1176. doi: 10.3389/fphys.2019.01176, PMID: 31572221 PMC6753868

[ref47] Pagaduan J. C. Wu S. S. X. Fell J. W. Chen Y.-S. (2021). Effect of acute heart rate variability biofeedback on H-reflex modulation: a pilot study. J. Hum. Kinet. 76, 83–88. doi: 10.2478/hukin-2021-0001, PMID: 33603926 PMC7877276

[ref48] Penttilä J. Helminen A. Jartti T. Kuusela T. Huikuri H. V. Tulppo M. P.. (2001). Time domain, geometrical and frequency domain analysis of cardiac vagal outflow: effects of various respiratory patterns. Clin. Physiol. 21, 365–376. doi: 10.1046/j.1365-2281.2001.00337.x, PMID: 11380537

[ref49] Petersen S. E. Posner M. I. (2012). The attention system of the human brain: 20 years after. Annu. Rev. Neurosci. 35, 73–89. doi: 10.1146/annurev-neuro-062111-150525, PMID: 22524787 PMC3413263

[ref50] Pizzoli S. F. M. Marzorati C. Gatti D. Monzani D. Mazzocco K. Pravettoni G. (2021). A meta-analysis on heart rate variability biofeedback and depressive symptoms. Sci. Rep. 11:6650. doi: 10.1038/s41598-021-86149-7, PMID: 33758260 PMC7988005

[ref51] Posner M. I. (1980). Orienting of attention. Q. J. Exp. Psychol. 32, 3–25. doi: 10.1080/00335558008248231 7367577

[ref52] Posner M. I. Petersen S. E. (1990). The attention system of the human brain. Annu. Rev. Neurosci. 13, 25–42. doi: 10.1146/annurev.ne.13.030190.000325 2183676

[ref53] Prinsloo G. E. Derman W. E. Lambert M. I. Rauch H. G. L. (2013a). The effect of a single session of short duration biofeedback-induced deep breathing on measures of heart rate variability during laboratory-induced cognitive stress: a pilot study. Appl. Psychophysiol. Biofeedback 38, 81–90. doi: 10.1007/s10484-013-9210-0, PMID: 23435801

[ref54] Prinsloo G. E. Derman W. E. Lambert M. I. Rauch H. G. L. (2013b). The effect of a single episode of short duration heart rate variability biofeedback on measures of anxiety and relaxation states. Int. J. Stress. Manag. 20, 391–411. doi: 10.1037/a0034777

[ref55] Prinsloo G. E. Rauch H. G. L. Karpul D. Derman W. E. (2013c). The effect of a single session of short duration heart rate variability biofeedback on EEG: a pilot study. Appl. Psychophysiol. Biofeedback 38, 45–56. doi: 10.1007/s10484-012-9207-0, PMID: 23129056

[ref56] Prinsloo G. E. Rauch H. G. L. Lambert M. I. Muench F. Noakes T. D. Derman W. E. (2011). The effect of short duration heart rate variability (HRV) biofeedback on cognitive performance during laboratory induced cognitive stress. Appl. Cognit. Psychol. 25, 792–801. doi: 10.1002/acp.1750

[ref57] Quintana D. S. Elvsåshagen T. Zak N. Norbom L. B. Pedersen P. Ø. Quraishi S. H.. (2017). Diurnal variation and twenty-four hour sleep deprivation do not Alter supine heart rate variability in healthy male young adults. PLoS One 12:e0170921. doi: 10.1371/journal.pone.0170921, PMID: 28151944 PMC5289546

[ref58] Ramírez E. Ortega A. R. Del Reyes Paso G. A. (2015). Anxiety, attention, and decision making: the moderating role of heart rate variability. Int. J. Psychophysiol. 98, 490–496. doi: 10.1016/j.ijpsycho.2015.10.007, PMID: 26555079

[ref59] Sammito S. Böckelmann I. (2016). Reference values for time- and frequency-domain heart rate variability measures. Heart Rhythm. 13, 1309–1316. doi: 10.1016/j.hrthm.2016.02.006, PMID: 26883166

[ref60] Schumann A. La Cruz F. Köhler S. Brotte L. Bär K.-J. (2021). The influence of heart rate variability biofeedback on cardiac regulation and functional brain connectivity. Front. Neurosci. 15:691988. doi: 10.3389/fnins.2021.691988, PMID: 34267625 PMC8275647

[ref61] Sevoz-Couche C. Laborde S. (2022). Heart rate variability and slow-paced breathing:when coherence meets resonance. Neurosci. Biobehav. Rev. 135:104576. doi: 10.1016/j.neubiorev.2022.104576, PMID: 35167847

[ref62] Sherlin L. Muench F. Wyckoff S. (2010). Respiratory sinus arrhythmia feedback in a stressed population exposed to a brief stressor demonstrated by quantitative EEG and sLORETA. Appl. Psychophysiol. Biofeedback 35, 219–228. doi: 10.1007/s10484-010-9132-z, PMID: 20414803

[ref63] Siepmann M. Aykac V. Unterdörfer J. Petrowski K. Mueck-Weymann M. (2008). A pilot study on the effects of heart rate variability biofeedback in patients with depression and in healthy subjects. Appl. Psychophysiol. Biofeedback 33, 195–201. doi: 10.1007/s10484-008-9064-z, PMID: 18807175

[ref64] Song H.-S. Lehrer P. M. (2003). The effects of specific respiratory rates on heart rate and heart rate variability. Appl. Psychophysiol. Biofeedback 28, 13–23. doi: 10.1023/A:1022312815649 12737093

[ref65] Sørensen L. Wass S. Osnes B. Schanche E. Adolfsdottir S. Svendsen J. L.. (2019). A psychophysiological investigation of the interplay between orienting and executive control during stimulus conflict: a heart rate variability study. Physiol. Behav. 211:112657. doi: 10.1016/j.physbeh.2019.112657, PMID: 31445015

[ref66] Szeska C. Richter J. Wendt J. Weymar M. Hamm A. O. (2020). Promoting long-term inhibition of human fear responses by non-invasive transcutaneous vagus nerve stimulation during extinction training. Sci. Rep. 10:1529. doi: 10.1038/s41598-020-58412-w, PMID: 32001763 PMC6992620

[ref67] Thayer J. F. Hansen A. L. Saus-Rose E. Johnsen B. H. (2009). Heart rate variability, prefrontal neural function, and cognitive performance: the neurovisceral integration perspective on self-regulation, adaptation, and health. Ann. Behav. Med. 37, 141–153. doi: 10.1007/s12160-009-9101-z, PMID: 19424767

[ref68] Thayer J. F. Lane R. D. (2000). A model of neurovisceral integration in emotion regulation and dysregulation. J. Affect. Disord. 61, 201–216. doi: 10.1016/s0165-0327(00)00338-4, PMID: 11163422

[ref69] Thayer J. F. Lane R. D. (2009). Claude Bernard and the heart-brain connection: further elaboration of a model of neurovisceral integration. Neurosci. Biobehav. Rev. 33, 81–88. doi: 10.1016/j.neubiorev.2008.08.004, PMID: 18771686

[ref70] Tinello D. Kliegel M. Zuber S. (2022). Does heart rate variability biofeedback enhance executive functions across the lifespan? A Systematic Review. J. Cogn. Enhanc. 6, 126–142. doi: 10.1007/s41465-021-00218-3, PMID: 35299845 PMC8901517

[ref71] Vaschillo E. G. Vaschillo B. Lehrer P. M. (2006). Characteristics of resonance in heart rate variability stimulated by biofeedback. Appl. Psychophysiol. Biofeedback 31, 129–142. doi: 10.1007/s10484-006-9009-3, PMID: 16838124

[ref72] Voss A. Heitmann A. Schroeder R. Peters A. Perz S. (2012). Short-term heart rate variability--age dependence in healthy subjects. Physiol. Meas. 33, 1289–1311. doi: 10.1088/0967-3334/33/8/1289 22813869

[ref73] Vuilleumier P. (2009). “The role of the human amygdala in perception and attention” in The human amygdala. eds. Whalen P. J. Phelps E. A. (New York, NY: The Guilford Press), 220–249.

[ref74] Wells R. Outhred T. Heathers J. A. J. Quintana D. S. Kemp A. H. (2012). Matter over mind: a randomised-controlled trial of single-session biofeedback training on performance anxiety and heart rate variability in musicians. PLoS One 7:e46597. doi: 10.1371/journal.pone.0046597, PMID: 23056361 PMC3464298

[ref75] Wendt J. König J. Hufenbach M. C. Koenig J. Thayer J. F. Hamm A. O. (2019). Vagally mediated heart rate variability and safety learning: effects of instructions and number of extinction trials. Psychophysiology 56:e13404. doi: 10.1111/psyp.13404, PMID: 31149740

[ref76] You M. Laborde S. Salvotti C. Zammit N. Mosley E. Dosseville F. (2022). Influence of a single slow-paced breathing session on cardiac vagal activity in athletes. Int. J. Ment. Heal. Addict. 20, 1632–1644. doi: 10.1007/s11469-020-00467-x

[ref77] Zaccaro A. Piarulli A. Laurino M. Garbella E. Menicucci D. Neri B.. (2018). How breath-control can change your life: a systematic review on psycho-physiological correlates of slow breathing. Front. Hum. Neurosci. 12:353. doi: 10.3389/fnhum.2018.00353, PMID: 30245619 PMC6137615

[ref78] Zaccaro A. Piarulli A. Melosini L. Menicucci D. Gemignani A. (2022). Neural correlates of non-ordinary states of consciousness in pranayama practitioners: the role of slow nasal breathing. Front. Syst. Neurosci. 16:803904. doi: 10.3389/fnsys.2022.803904, PMID: 35387390 PMC8977447

[ref79] Zahn D. Adams J. Krohn J. Wenzel M. Mann C. G. Gomille L. K.. (2016). Heart rate variability and self-control--a meta-analysis. Biol. Psychol. 115, 9–26. doi: 10.1016/j.biopsycho.2015.12.007 26747415

[ref80] Zhang J. (2007). Effect of age and sex on heart rate variability in healthy subjects. J. Manip. Physiol. Ther. 30, 374–379. doi: 10.1016/j.jmpt.2007.04.001 17574955

